# Responses to Elevated c-di-GMP Levels in Mutualistic and Pathogenic Plant-Interacting Bacteria

**DOI:** 10.1371/journal.pone.0091645

**Published:** 2014-03-13

**Authors:** Daniel Pérez-Mendoza, Isabel M. Aragón, Harold A. Prada-Ramírez, Lorena Romero-Jiménez, Cayo Ramos, María-Trinidad Gallegos, Juan Sanjuán

**Affiliations:** 1 Dpto. Microbiología del Suelo y Sistemas Simbióticos, Estación Experimental del Zaidín, CSIC, Granada, Spain; 2 Área de Genética, Universidad de Málaga, Instituto de Hortofruticultura Subtropical y Mediterránea “La Mayora”, Universidad de Málaga-CSIC (IHSM-UMA-CSIC), Málaga, Spain; Universidad Pública de Navarra, Spain

## Abstract

Despite a recent burst of research, knowledge on c-di-GMP signaling pathways remains largely fragmentary and molecular mechanisms of regulation and even c-di-GMP targets are yet unknown for most bacteria. Besides genomics or bioinformatics, accompanying alternative approaches are necessary to reveal c-di-GMP regulation in bacteria with complex lifestyles. We have approached this study by artificially altering the c-di-GMP economy of diverse pathogenic and mutualistic plant-interacting bacteria and examining the effects on the interaction with their respective host plants. Phytopathogenic *Pseudomonas* and symbiotic *Rhizobium* strains with enhanced levels of intracellular c-di-GMP displayed common free-living responses: reduction of motility, increased production of extracellular polysaccharides and enhanced biofilm formation. Regarding the interaction with the host plants, *P. savastanoi* pv. savastanoi cells containing high c-di-GMP levels formed larger knots on olive plants which, however, displayed reduced necrosis. In contrast, development of disease symptoms in *P. syringae*-tomato or *P. syringae*-bean interactions did not seem significantly affected by high c-di-GMP. On the other hand, increasing c-di-GMP levels in symbiotic *R. etli* and *R. leguminosarum* strains favoured the early stages of the interaction since enhanced adhesion to plant roots, but decreased symbiotic efficiency as plant growth and nitrogen contents were reduced. Our results remark the importance of c-di-GMP economy for plant-interacting bacteria and show the usefulness of our approach to reveal particular stages during plant-bacteria associations which are sensitive to changes in c-di-GMP levels.

## Introduction

The permanent exchange of multiple signals between plant and bacteria during the establishment of pathogenic or mutualistic interactions need to be integrated to coordinate, in time and space and upon the local environmental conditions, the expression of essential determinants allowing colonization and eventually invasion of the host. Bacterial motility and chemotaxis, exopolysaccharide (EPS) production, biofilm formation and secretion of adhesion and effector proteins are key and very often shared traits among bacteria that interact with plants, both mutualistic and pathogenic [1]–[3]. Complex regulatory networks involving inter- and intracellular signaling, orchestrate fine-tuning of all these bacterial traits.

In the last decade cyclic-di-GMP (c-di-GMP) has emerged as an ubiquitous second messenger in bacteria. Initially identified as an allosteric activator of bacterial cellulose synthase [4], this second messenger can regulate biofilm formation, motility, the cell cycle, virulence and other important morphological and cellular processes in bacteria, playing a key role in the switch between motile and sedentary lifestyles ( [5] and references therein). Despite a recent burst of research in this field, knowledge on c-di-GMP signaling pathways remains largely fragmentary and molecular mechanisms of regulation and even c-di-GMP targets are yet unknown for most bacteria [5].

The c-di-GMP is synthesized from two molecules of GTP by the action of diguanylate cyclases (DGC), and hydrolyzed by specific phosphodiesterases (PDE). GGDEF protein domains function in c-di-GMP synthesis, whereas the c-di-GMP PDE activity is associated with EAL or HD-GYP domains [6]–[9]. Bacterial genomes usually encode several and very often up to dozens proteins that synthesize or hydrolize c-di-GMP, which contrasts with the relatively low number of known effector molecules whose activity and/or stability is modulated upon binding to c-di-GMP. This secondary messenger binds to diverse classes of structurally and functionally unrelated proteins (like enzymes or transcription factors) and even to RNAs (riboswitches, reviewed in [10]). Thus, c-di-GMP can act at the transcriptional, posttranscriptional and posttranslational levels. Moreover, it has been proposed that distinct c-di-GMP signaling modules are separated in time and/or space within the cell [11]. The likely functional redundance among c-di-GMP metabolizing proteins, together with the presumed complexity of c-di-GMP signaling very often hinder genetic and genomic approaches to reveal c-di-GMP networks and components affecting a particular function, especially in bacteria with varying lifestyles like those interacting with plants. Thus, alternative approaches like the artificial alteration of the cellular c-di-GMP economy allows focusing on particular c-di-GMP functional targets. It has been reported that the overproduction of GGDEF domain proteins very often triggers phenotypes related to sessility, like the synthesis of adhesins and biofilm matrix components, whereas an excess production of proteins with EAL domains usually causes the opposite phenotypes [12]–[18]. We have exploited the overexpression of a well characterized diguanylate cyclase to artificially raise the intracellular levels of this second messenger in diverse plant-interacting bacteria such as phytopathogenic *Pseudomonas* and symbiotic *Rhizobium* bacteria. The impact of elevated c-di-GMP levels on the virulence of pathogens and on root nodule formation by mutualistic rhizobia was examined. This type of approach will facilitate dissecting novel c-di-GMP functional targets during the establishment of plant-bacteria associations.

## Materials and Methods

### Bacteria and Culture Conditions

Bacterial strains and plasmids used in this work are listed in Table 1.

**Table 1:** Bacterial strains and plasmids used in this study.

Strains	Relevant characteristic	Reference or source
**Rhizobia strains**		
*R. etli* CFN42	Wild-type	[77]
*R. leguminosarum* bv. viciae UPM791	Wild-type, Sm^r^ derivative of 128C53	[78]
RetΔcelAB	CFN42 Δ*celAB*	This work
**Pseudomonas strains**		
*P. syringae* pv. tomato DC3000	Rif^r^	[79]
PtoΔwssBC	DC3000 Δ*wssBC*	This work
*P. syringae* pv. phaseolicola 1448A	Race 6	[80]
*P. savastanoi*	NCPPB 3335	[37]
***Escherichia coli*** ** strains**		
DH5α	*supE44*, Δ*lacU169*, Φ80, *lacZ*ΔM1, *recA1*, *endA1*, *gyrA96*, *thi1*, *relA1*, *5hsdR171*	[81]
S17.1	Tmp^r^, Sm^r^, Sp^r^; *thi*, *pro*, *recA*, *hsdR*, *hsdM*, Rp4Tc::Mu, Km::Tn*7*	[82]
β2163	(F−) RP4-2-Tc::Mu *_dapA*::(*erm-pir*) [KmR EmR]	[24]
OmniMAX	*F′* [*pro*AB*+ lacIq lac*ZΔM15 *Tn10(TetR)* Δ*(ccdAB)*] *mcr*A Δ*(mrr-hsdRMS-mcr*BC*) φ*80*(lacZ)*ΔM15Δ*(lacZ*YA*-argF)* U169 *end*A1 *rec*A1 *sup*E44 *thi-1 gyr*A96 *rel*A1 *ton*A *pan*D	Invitrogen
**Plasmids**		
pRP89	Ap^r^, pET11 derivate carrying pleD*, a mutant variant of *pleD* from *C. crescentus*	[7]
pJB3Tc19	Ap^r^, Tc^r^; cloning vector, P*_lac_* promoter	[22]
pJBpleD*	Ap^r^, Tc^r^; pJB3Tc19 derivate bearing a 1423 bp XbaI/EcoRI fragment containing pleD*	This work
pK18*mobsacB*	Km^r^; mobilizable suicide plasmid	[26]
pK18ΔcelAB	Km^r^; pK18*mobsacB* carrying the deleted version of the *celAB* genes	This work
pK18ΔwssBC	Km^r^; pK18*mobsacB* carrying the deleted version of the *wssBC* genes	This work
pCR-XL-TOPO	Km^r^; cloning vector for PCR products	Invitrogen
TOPOΔcelAB	Km^r^; pCR-XL-TOPO carrying the deleted version of the *celAB* genes	This work
TOPOΔwssBC	Km^r^; pCR-XL-TOPO carrying the deleted version of the *wssBC* genes	This work

Ap^r^, Km^r^, Nal^r^, Rif^r^, Sm^r^, Sp^r^, Tc^r^, Tmp^r^, stand for resistance to ampicillin, kanamycin, nalidixic acid, rifampicin, streptomycin, spectinomycin, tetracycline and trimethropin, respectively. NCPPB, National Collection of Plant Pathogenic Bacteria.

Overnight cultures of *Pseudomonas* strains (Pto, Pph, and Psv) were routinely grown in Luria–Bertani broth (LB; containing 10 g/L tryptone, 5 g/L yeast extract, 5 g/L NaCl) at 25°C. Starting cultures of rhizobial strains (Ret and Rle) were grown overnight at 28°C on TY broth (tryptone-yeast extract-CaCl_2_ [19]). MM medium [20] was used for both rhizobial and *Pseudomonas* strains in different assays, supplemented with 50 mM glucose for the latest. In some cases, MMF medium was used for *Pseudomonas* [21]. When required, antibiotics and other compounds were added at the following final concentrations: Tetracycline (Tc), 10 µg ml^−1^ (5 µg ml^−1^ for Ret); Congo Red (CR), 50 µg ml^−1^, Calcofluor (CF) 200 µg ml^−1^ (in solid media) or 100 µg ml^−1^ (in liquid media). All free-living assays with strains carrying pJB3Tc19 or pJBpleD* plasmids were done in media supplemented with Tc to prevent plasmid losses. To determine possible effects of plasmid pJBpleD* on growth rates, growth curves were performed and compared with strains carrying the empy vector pJB3Tc19. Overnight cultures were diluted to an OD_600_ of 0.05 in 100 ml Erlenmeyer flasks (3 replicates per strain) containing 25 ml of LB (*Pseudomonas*) or TY (*Rhizobium*) supplemented with Tc. Cultures were incubated in rotary shaker (180 r.p.m.) at 28°C and OD_600_ measured every 2–3 hours.

Stability of pJB3Tc19 or pJBpleD* plasmids in the absence of antibiotics was also determined. Overnight cultures grown under Tc selection were diluted 1/100 in nonselective LB (*Pseudomonas*) or TY (*Rhizobium*) media, and incubated for 12–24 h at 28°C with shaking. The cultures were again diluted in nonselective medium and the procedure repeated until the total number of generations reached at least 100. After this, serial dilutions were spread on non selective and selective agar plates, and CFUs (colony formig units) counted after incubation at 28°C. Stability was determined as the ratio (%) of CFUs appeared in selective medium of the total CFUs in nonselective plates.

### Recombinant DNA Techniques

Molecular biology techniques were performed according to standard protocols and manufacturer’s instructions. Plasmid pJBpleD* was constructed by subcloning the XbaI/EcoRI fragment containing pleD* gene from pRP89 plasmid [7] into the broad host range vector pJB3Tc19 [22] previously digested with the same restriction enzymes. Transformation of *Pseudomonas* strains with the different plasmid constructions was carried out by electroporation. Electro-competent cells were prepared according to [23]. Briefly, cells were mixed with DNA (0.3–0.5 µg of DNA per ml of cell suspension), transferred to 0.1 cm cuvettes and submitted to a high-voltage pulse (1.800 V) for 5 ms by using a MicroPulser electroporation apparatus (Bio-Rad). Transformants were selected in LB agar plates supplemented with tetracycline. Plasmid pJBpleD* and pJB3Tc19 were introduced in rhizobia strains by bacterial conjugation using the *E. coli* β2163 donor strain [24] in matings as previously described [25].

### Construction of Cellulose Synthase Mutants

To generate a *R. etli* CFN42 derivative carrying a deletion of cellulose synthase *celAB* genes (RHE_CH01542 and RHE_CH01543), two fragments flanking the two ORFs were amplified separately, using two pairs of primers celAB-1/celAB-2 (836 bp) and celAB-3/celAB-4 (969 bp, Table S1). These two fragments were purified and used as template DNA for an overlapping PCR with primers celAB-1 and CelAB-4. The final fragment was cloned into pCR-XL-TOPO (Invitrogen) and sequenced. A correct *Hind*III/*Xba*I DNA insert was isolated and subcloned into the suicide plasmid pK18*mobSacB* [26] generating the pK18ΔcelAB. The pK18ΔcelAB plasmid was introduced into *R. etli* CFN42 by a biparental conjugation from *E. coli* S17.1 strain, and the *celAB* deletion of 3277 bp (1618863–1622139) was generated by homologous recombination, following procedures described in [26]. The Ret ΔcelAB mutant contained an untagged (without resistance or reporter genes) deletion of both ORFs that was corroborated by PCR.

For construction of PtoΔwssBC mutant, two fragments including the upstream region of *wssB* and the downstream region of *wssC* genes from strain DC3000 were amplified separately using two pairs of primers: 1026-F/1027-R (1192 bp) and 1028-F/1029-R (1311 bp), respectively (Table S1). These two fragments were purified and used as a DNA template for an overlapping PCR in combination with primers 1026-F and 1029-R. The final fragment was cloned into pCR-XL-TOPO (Invitrogen) and sequenced. A *EcoR*I DNA fragment was purified and subcloned into the *EcoR*I site of the suicide plasmid pK18*mobsacB* [26] generating the pK18ΔwssBC. The pK18ΔwssBC plasmid was introduced into Pto by electroporation, and the *wssBC* deletion of 2642 bp was generated by homologous recombination according to [26]. The PtoΔwssBC mutant was corroborated by PCR.

### Preparation of mRNA and Quantitative RT-PCR Assay

Pto DC3000, Pph 1448A and Psv NCPPB 3335 carrying plasmids pJB3Tc19 and pJBpleD* were grown until OD_600_ of 0.5 in LB medium supplemented with the appropriate antibiotics at 20°C. Cells were induced in MMF by 3 hours at 20°C. Then, cells were pelleted and processed for RNA isolation using TRI Reagent LS (Molecular Research Center, Cincinnati, OH, USA) according to the manufacturer’s instructions, except that the TRI Reagent was preheated at 70°C and the lysis step was carried out at 65°C. The RNA obtained was treated with TURBO DNA-free™-Kit (Applied Biosystems; California, USA). RNA concentration was determined spectrophotometrically and its integrity was assessed by agarose gel electrophoresis.

DNA-free total RNA (1 µg) was retrotranscribed to cDNA with the iScript cDNA synthesis kit (BioRad; California, USA) using random hexamers. The specific primer pairs used to amplify cDNA are shown in Table S1. Primer efficiency, qRT-PCR’s and confirmation of the specificity of the amplification reactions were performed as described in [27]. Relative transcript abundance was calculated using the ΔΔCt method [28]. Transcriptional data were normalized to the housekeeping gene *gyrA* using Bio-Rad i5 software v.2.1. The expression of a given gene relative to *gyrA* was calculated as previously described in [27].

### Motility Assays

For swimming motility assays performed with rhizobia, bacteria were grown on MM plates for 48 hours, resuspended in MM and adjusted to an OD_600_ of 1. Two µl aliquots were spotted onto semisolid Bromfield (BM) medium (0.04% tryptone, 0.01% yeast extract, 0.3% Agar and 0.01% CaCl_2_·2H_2_O) and halo diameters measured after incubation for 3 days at 28°C. Surface motility with rhizobia was analyzed using a protocol previously described [29], in which 2 µl of cultures grown in TY (OD_600_ = 1), washed in MM and resuspended in 0.1 volume of the latter medium, were dropped into swarm plates and allowed to dry for 10 min. Swarm plates were prepared with 20 ml of MM containing 0.6% purified agar (Agar Noble, Difco), and air dried at room temperature for 15 min. Incubation periods of 3 days at 28°C were enough to observe surface motility. Migration zones were calculated as the average length of the two sides of a rectangle able to exactly frame each colony. For swimming motility assays with *Pseudomonas* strains, bacteria were grown on LB plates for 48 hours, resuspended in 10 mM MgCl_2_ and adjusted to an OD_660_ of 2. Two µl aliquots were inoculated in the centre of 0.3% agar LB plates with Tc and incubated 48 hours at 25°C. The diameters of the swimming halos were measured after 24 and 48 hours on LB plates. Three motility plates were used for each strain and the experiment was repeated with three independent cultures. For swarming assays, the cell suspensions were prepared as above but the 2 µl aliquots were deposited on the top of PG-agar plates (0.5% proteose peptone No. 3 (Difco 212693) and 0.2% glucose with 0.5% Difco Bacto-Agar) with Tc and incubated at 25°C. The surface motility was observed after 24 hours. Three motility plates were used for each strain, and the experiment was repeated with three independent cultures.

### Calcofluor Binding Assays

Quantification of the exopolysaccharides CF^+^ of the different strains were performed as follows: *Pseudomonas* strains were refreshed on LB plates with respective antibiotics. Bacteria were suspended in 3 ml of 10 mM MgCl_2_ and diluted into 10 ml flasks containing MM supplemented with CF (100 µM final concentration) up to initial OD_600_ = 0.05 and incubated at 20°C under agitation for 24 h.

Starting cultures of rhizobial strains were prepared as detailed above. After washing with MM, cultures were diluted 1/100 into 10 ml flasks containing MM supplemented with CF (100 µM final concentration) at 28°C under agitation for 48 h.

Afterwards, cultures of the different strains were centrifuged for 10 minutes at 4000 rpm. Supernatant containing broth with unbound CF of each biological replicate was removed; the pellet was suspended in 2 ml of distilled water and disposed in wells of 24-well plates. Similar growth of all strains was confirmed and CF binding measurements for 3 biological replicates of each strain were performed in a PTI fluorimeter (Photon Technology International), expressing the results in arbitrary units ± standard error.

### Biofilm Assays

Starting cultures of rhizobial strains were prepared as above. After washing with MM, cultures were diluted up to OD_600_ = 0.1 in sterile MM. 200 µl samples of diluted strains were aliquoted into the wells of sterile 96-well polystyrene plates (Sarstedt) and were left in a humid environment at 28°C for 3 days. After incubation, the liquid from the wells was removed by aspiration and wells were washed with 240 µl of deionised water. 240 µl of crystal violet (0.1% in water) was added to each well and left to stain for 1 hour. The crystal violet was removed by aspiration and each well was washed carefully with 3×240 µl of deionised water. 240 µl of 70% ethanol was added to each well and the plate was gently agitated for at least 1 hour. The purple colour of appropriate dilutions was quantified by measurement of A_550_ in a Sunrise microplate reader (Tecan).

Starting cultures of *Pseudomonas* strains were carried out from plates as described above and 4 ml of diluted cultures in MM [20] supplemented with 50 mM glucose (OD_600_ = 0.05) and Tc were disposed in glass tubes and let grow under static conditions at 20°C for 72 h. A representative pellicle formed in the Air-Liquid interface from each strain was imaged. The same procedure was followed when the assays were performed in non-treated 24-well polystyrene plates (Iwaki).

### Intracellular c-di-GMP Measurements

c-di-GMP was extracted using a protocol based on a previous report [30]. Three biological replicates of each strain were grown in 10 ml of TY for rhizobia or LB broth for *Pseudomonas* strains. Formaldehyde at a final concentration of 0.19% was added and the cells harvested by centrifugation (10 min at 4000 rpm). The pellet was washed in 1 ml of iced deionised water, centrifuged for 3 min at 13000 rpm, then resuspended in 0.5 ml of iced deionised water and heated to 95°C for 5 min. A volume of 925 µl of iced absolute ethanol was added to reach a final concentration of 65%. Nucleotides were extracted by 30 sec of vortex followed by a centrifugation step (3 min at 13000 rpm). Supernatants containing extracted nucleotides were then evaporated to dryness at 50°C in a speed-vacuum system. The pellet was resuspended by vigorous vortexing in 300 µl of AcNH_4_ 10 mM (pH 5.5). Samples were filtered through 0.45 µm GHP membranes (GHP Acrodisc, PALL). Samples were spiked at a concentration of 250 nM by mixing 100 µl of a solution of 750 nM of synthetic of c-di-GMP (Axxora) disolved in AcNH_4_ 10 mM pH 5.5 with 200 µl of each sample. Samples were analysed by reversed phase-coupled HPLC-MS/MS. High performance liquid chromatography was performed on an Agilent 1100 coupled to a 3×125 mm column Waters Spherisorb 5 µm ODS2 (C-18). Running conditions were optimized using synthetic c-di-GMP as a reference. ESI-MS mass spectra were measured on an Esquire 6000 (Bruker Daltonics) and on a TSQ7000 (Finnigan) mass spectrometer. Matrix-assisted laser desorption ionization spectra were measured on a Reflex III spectrometer (Bruker Daltonics). To confirm the identity of the substance, relevant peaks were fragmented by ESI-MS using the positive ion mode. Three major ions were visible in the c-di-GMP fragmentation pattern, and m/z 152 and 540 corresponded to products from single bond fragmentation, and 248 originated from double bond fragmentations. The area of the ion m/z 540 peak was used to estimate the amount of c-di-GMP in each sample (similar values were obtained with the other ions; data not shown).

For quantification, a standard curve was established using synthetic c-di-GMP (Axxora) dissolved in ammonium acetate (10 mM pH 5.5) at a range of concentrations (20 nM, 200 nM, 2 µM and 20 µM). After subtracting the basal 250 nM spike, c-di-GMP concentrations in each strain culture were standardised with the total protein content, determined by Bradford assay [31].

### Alginate Quantification

For quantification of bacterial alginate production, three-day plate cultures of *P. syringae* on MM medium supplemented with 50 mM glucose at 20°C were resuspended in 0.9% NaCl for alginate extraction and quantification as in [32]. Alginate levels of each strain were standardised by total protein of the initial sample determined by Bradford assay [31] expressed in µg uronic acids/mg of total cell protein ± standard deviation.

### Plant Root Binding Assays

Bean and vetch seeds (*Phaseolus vulgaris* cv. contender and *Vicia sativa* cv. Jose, respectively) were surface-sterilized by washing the seeds abundantly with tap water followed by treatment (2×5 minutes) with ethanol 100%. Ethanol was removed by washing three times with deionised water and a second treatment was applied by adding sodium hypochlorite 5% for 5 minutes. Seeds were washed with abundant sterile deionised water to remove any remains of sodium hypochlorite. Bean seeds were germinated in purified agar:water 1% for 72 h at 28°C in the dark. Vetch seeds were soaked in sterilised water for 10 h and then placed on plates with water wet filter paper at 28°C for 48 h in the dark.

Nine seedlings were transferred into a flask containing 10^8^ cells of each strain in 50 ml of nutrient solution and gently agitated at 28°C for 4 h. After this time, roots were separated from the shoots and 3 groups of 3 roots each were separately taken and washed vigorously four times with 10 ml of sterile deionised water in Falcon tubes, to removed unbound or weakly bound bacteria. After these washings, 10 ml of MM with 2 mM EDTA were added and root bound bacteria were released by 2 cycles of 1 min of vortex and 1 min of sonication. Serial dilutions were prepared and spread onto Congo red MM tetracycline plates and incubated at 28°C for 3–4 days. CFUs were counted to determine bound rhizobial cells harbouring pJBpleD* (red colonies) or the empty vector pJB3Tc19 (white colonies). The ratio (%) of bounded cells per g of root to total inoculum cells were calculated for each strain.

### Rhizobia Symbiotic Assays

Bean (*Phaseolus vulgaris* cv. Contender) and vetch (*Vicia sativa* cv. Jose) seeds were surface-sterilized and germinated as above. To test the infectivity of rhizobial strains, 12 bean or 25 vetch seedlings were sown in Leonard-type assemblies containing a mix (3∶1) vermiculite:perlite on the top part and nitrogen-free nutrient solution [33] in the bottom. Each seedling was inoculated with 10^6^ CFU of the compatible bacterial symbiont (Ret for beans and Rle for vetch). Bean plants were cultivated in a growth chamber with 16/8-h light/dark photoperiod at 24/16°C day/night and 75% relative humidity. Vetch plants were grown in a greenhouse.

The shoot fresh weight and the number of nodules were determined after 29 days for bean and 41 days for vetch plants, respectively. Shoot dry weights were determined after desiccating the samples in an oven (65°C) for 3 days. Dry shoots were ground and total nitrogen contents were determined following the Dumas method at the Ionomics Service of the institute CEBAS-CSIC (Murcia, Spain; http://www.cebas.csic.es/general_english/ionomics.html). To evaluate the presence or absence of nodule bacteria carrying pJBpleD* or pJB3Tc19 plasmids, fifty of the nodules formed by each strain were surface-sterilised with HgCl_2_ 0.25% for 5 min followed by washing with abundant sterile deionised water. Nodules were individually crushed and the content spread on TY and TY+Tc plates. Statistical data analysis was performed by analysis of variance followed by Anova test (P≤0.05) with IBM SPSS Statistic 20 software.

### Pseudomonas Virulence Assays

Seeds of *Solanum lycopersicum* cv. Moneymaker and *Phaseolus vulgaris* cv. Canadian Wonder were germinated and grown in a growth chamber with 16/8-h light/dark photoperiod at 24/18°C day/night and 70% relative humidity for 5 weeks or 2 weeks, respectively.

*P. syringae* pv. tomato DC3000 and pv. phaseolicola 1448A grown on LB plates for 48 h at 28°C, were suspended in 10 mM MgCl_2_ and the inocula adjusted to 10^8^ cfu/ml or 10^5^ cfu/ml. Infiltration was achieved by pressing the bacterial suspension into the abaxial side of the leaf using a syringe without needle. For symptom observation and bacterial population counting in plant, individual inocula (10^6^ cfu/ml) of the strains were sprayed into different plants and sampling were performed 3 hours after inoculation (time 0) and several days after inoculation (dpi, days post-inoculation) to determine output CFU. Bacteria were recovered from the infected leaves using a 10-mm-diameter cork-borer. Five disks (3.9 cm^2^) per plant were homogenized by mechanical disruption into 1 ml of 10 mM MgCl_2_ and counted by plating serial dilutions onto LB plates with the corresponding antibiotics.

Olive plants (*Olea europaea* L.) derived from seeds germinated *in vitro* (originally collected from a cv. Arbequina plant) were micropropagated, rooted and maintained as previously described [34], [35].

Micropropagated olive plants were infected in the stem wound with bacterial suspension (approximately 10^3^ CFU) and incubated for 30 days in a growth chamber as described by [34]. The morphology of the olive plants infected with bacteria was visualized using a stereoscopic microscope Leica MZ FLIII (Leica Microsystems, Wetzlar, Germany). To analyse the pathogenicity of *P. savastanoi* pv. savastanoi isolates in 1-year-old olive explants (woody plants), micropropagated olive plants were transferred to soil and maintained in a greenhouse at 27°C with a relative humidity of 58% under natural daylight. The plants were wounded in the stem with a sterile scalpel and infected with approximately 10^6^ CFU of *P. savastanoi* pv. savastanoi as previously described [36], [37]. The wounds in inoculated plants, which were 0.5 cm deep and spanned from the stem surface to the cambial area, were protected with Parafilm M (American National Can, Chicago, IL, USA) for one week post-inoculation. Morphological changes, scored at 90 dpi, were captured with a high-resolution digital camera Nikon DXM 1200 (Nikon Corporation, Tokyo, Japan). Knot volume was calculated by measuring length, width (subtracting the stem diameter measured above and under the knot from that measured below the knot) and height of the knot with a Vernier caliper [38], [39]. Statistical data analysis was performed by analysis of variance followed by t-Student test (P≤0.05). For counting bacterial populations, bacteria were recovered from the knots at different times post-inoculation using a mortar containing 1 ml of 10 mM MgCl_2_, and serial dilutions were plated onto LB medium (to detect the total amount of Psv cells) and LB-Tc medium (to detect bacterial cells maintaining the PleD* plasmid). Population densities were calculated from at least three independent replicates.

## Results

### Increasing the Intracellular Levels of c-di-GMP

To increase the intracellular levels of c-di-GMP in our model bacteria, we used the PleD* protein, a constitutively active DGC variant of the well-characterised DGC PleD from *Caulobacter crescentus* [40]. The pleD* gene was cloned under the control of the *lac* promoter in a plasmid vector, pJB3Tc19, that can replicate in both rhizobia and *Pseudomonas* strains. The resulting construction pJBpleD* was introduced in the model strains *Rhizobium etli* CFN42 (Ret) and *Rhizobium leguminosarum* bv. viciae UPM791 (Rle), and *Pseudomonas savastanoi* pv. savastanoi NCPPB 3335 (Psv) and *Pseudomonas syringae* [pv. phaseolicola 1448A (Pph) and pv. tomato DC3000 (Pto)] and intracellular c-di-GMP contents were quantified. The over-expression of pleD* generated strong c-di-GMP increases in all the strains compared to control strains containing the empty vector pJB3Tc19, being higher in rhizobial strains than in the *Pseudomonas* (Fig. S1).

### Stability of Plasmid pJBpleD* and Effects on Growth Rates

We determined whether pleD* expression and high c-di-GMP intracellular levels could affect bacterial growth rates in laboratory media. However, formation of flocs induced by pleD* (see below) hindered measuring culture ODs and even countingCFU. Pto and Ret mutants impaired in cellulose production, which did not show such aggregative behaviour in rich medium (see below), were used for this purpose. However, no significant changes in growth rates were observed for the Pto or Ret Cel^−^ strains carrying the pleD* plasmid (Figure S2). Likewise, we did not observe any growth delay of Cel^+^ strains carrying pJBpleD* on agar plates compared to strains carrying the empty vector.

Although all free-living assays with pJBpleD* strains were done in selective media supplemented with Tc, we were also interested to know plasmid stability under nonselective conditions. Plasmid stability in the absence of Tc, was variable and highly dependant on the bacterial strain. In all *Pseudomonas* strains both pJB3Tc19 and pJBpleD* plasmids were similarly stable (Table S2). In *R. leguminosarum* stability of both the vector and the pJBpleD* plasmid was also similar and relatively high. In contrast, only 8.3% of *R. etli* CFUs were able to maintain the pJB3Tc19 vector whereas only 0.6% maintained pJBpleD* after approximately 100 generations (Table S2).

### Effects of Increased c-di-GMP Levels on Free-living Phenotypes

**Figure 1:**
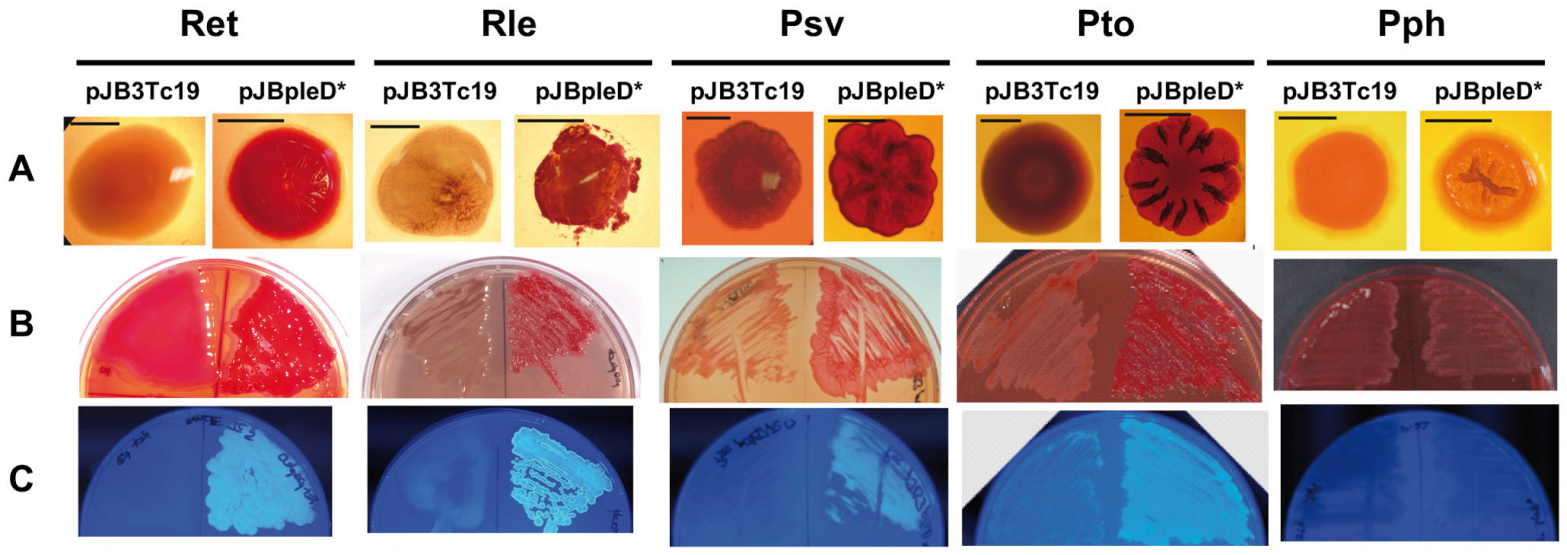
Effects of high c-di-GMP on bacterial external features. Colony morphology (**A**) and Congo red (**B**) or Calcofluor staining (**C**) of *Rhizobium etli* CFN42 (Ret), *Rhizobium leguminosarum* bv. viciae UPM791 (Rle), *Pseudomonas savastanoi* pv. savastanoi NCPPB 3335 (Psv), *Pseudomonas syringae* pv. tomato DC3000 (Pto) and *Pseudomonas syringae* pv. phaseolicola 1448 (Pph) expressing pleD* (pJBpleD*) and their respective control strains (pJB3Tc19, empty vector). Colonies were imaged after growth under the following conditions: for Ret and Rle, 3 days at 28°C in MM or YGT plates, respectively, supplemented with tetracycline (10 µg/ml or 5 µg/ml for Rle and Ret, respectively), CR (125 µg/ml; **A** and **B**) or CF (200 µg/mL; **C**). For Psv, Pto and Pph, 3 days at 20°C in MM (supplemented with 15% of glycerol for Pph) agar plates, supplemented with tetracycline (10 µg/ml) and with CR (125 µg/ml; **A** and **B**) or CF (200 µg/mL; **C**). Scale bars in (**A**) correspond to 1 mm.

Over-expression of PleD* generated wrinkled colonies that stained in media with Congo Red (CR^+^) in all strains, except in Pph that showed no significant binding to CR (Fig. 1). PleD* also increased 5- to 80-fold the Calcofluor (CF)-derived fluorescence of all the strains except Pph, which showed no significant differences and very low CF binding (Fig. S3).

Enhanced CR and CF binding suggested the production of cellulose in response to elevated c-di-GMP levels. To verify this possibility, Pto and Ret cellulose synthase mutants were generated. As expected, cultures of the Pto Cel^−^ mutant (PtoΔwssBC) expressing pleD* had lost the ability to stain with CR or fluoresce in the presence of CF (Fig. S3). The Ret Cel^−^ mutant (RetΔcelAB) expressing PleD* showed reduced CR and CF staining in comparison with the wild-type, but still bound significantly these dyes, i.e. CF-derived fluorescence was about 50% of the wild-type (Fig. S3). This and other phenotypes (reduced flocculation and biofilm formation, see below) suggested that the Cel^−^ mutation was likely affecting cellulose production but the high c-di-GMP levels were yet inducing the production by *R. etli* of another compound(s) able to bind CR and CF.

**Figure 2:**
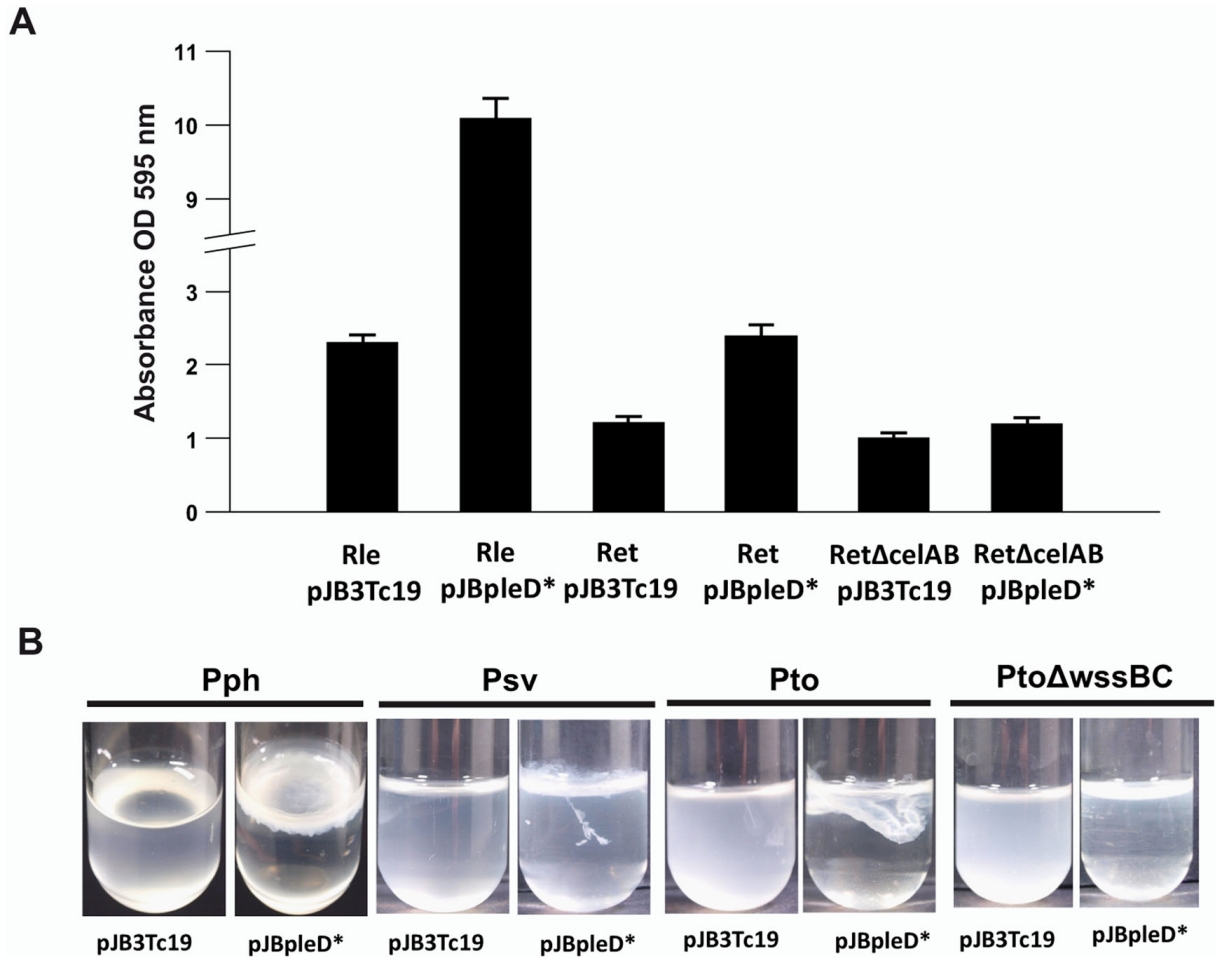
Effects of high c-di-GMP on biofilm production. Biofilm formation by *Rhizobium etli* CFN42 (Ret), *Rhizobium leguminosarum* bv. viciae UPM791 (Rle), *Pseudomonas savastanoi* pv. savastanoi NCPPB 3335 (Psv), *Pseudomonas syringae* pv. tomato DC3000 (Pto) and *Pseudomonas syringae* pv. phaseolicola 1448 (Pph) expressing pleD* (pJBpleD*) and their respective control strains (pJB3Tc19, empty vector). (**A**) Biofilm formation by Ret and Rle quantified after static growth, 72 h in MM in a 96-well plate at 28°C, by crystal violet (CV) staining and represented as the means of eight different wells for each strain ± standard error. Similar growth of all strains was confirmed by measuring OD_600_ before CV staining. (**B**) Air-Liquid biofilms produced by Pph, Psv, Pto expressing pleD* and their respective control strains after static growth in MM supplemented with 50 mM glucose and Tc, for 72 h at 20°C. Similar biofilms formed in microtitre plates.

A strong aggregative phenotype was observed when pleD* was over-expressed in rhizobial and *Pseudomonas* strains, with bacteria forming flocs after overnight culture, particularly in minimal media. Correlating with this, over-expression of pleD* in rhizobial strains (Ret and Rle) enhanced production of a strong and visually obvious biofilm that was easily quantified after crystal violet (CV) staining of microtitre plates (Fig. 2A). At least in the case of *R. etli*, this c-di-GMP induced biofilm required a cellulose synthase, since the Cel^−^ mutant had lost most of the PleD* enhanced biofilm capacity (Fig. 2A).

In contrast to rhizobia, over-expression of pleD* in the three *Pseudomonas* strains promoted the formation of an air-liquid (A-L) interface biofilm or pellicle maintained by bacterial attachment to the walls of the tubes (and microtitre wells) at the meniscus (Fig. 2B), instead of the formation of an archetypal biofilm attached to the container surface. These pellicles formed under static growth conditions and easily sank when gentle shaking was applied (Fig. 2B), what prevented quantification after CV staining. The Pto Cel^−^ mutant was still able to form pellicles similar to those of the wild type (Fig. 2B), strongly supporting that cellulose is not necessary for this type of biofilms. An analysis of the polysaccharides produced by Pto and Pph indicated that alginate was also present in the matrix of those pellicles. Alginate production was strongly induced by high c-di-GMP in both Pto and Pph, although total amounts of alginate were much higher in Pph (Fig. S4).

The presence of the pJBpleD* plasmid caused inhibition of both surface and swimming motilities in all the strains tested (see Fig. S5). In agreement with [37], Psv did not show any surface motility under different conditions, therefore it was not possible to evaluate the influence of PleD* on this feature.

### Influence of High Intracellular Levels of c-di-GMP on Rhizobia Interaction with Legumes

**Figure 3:**
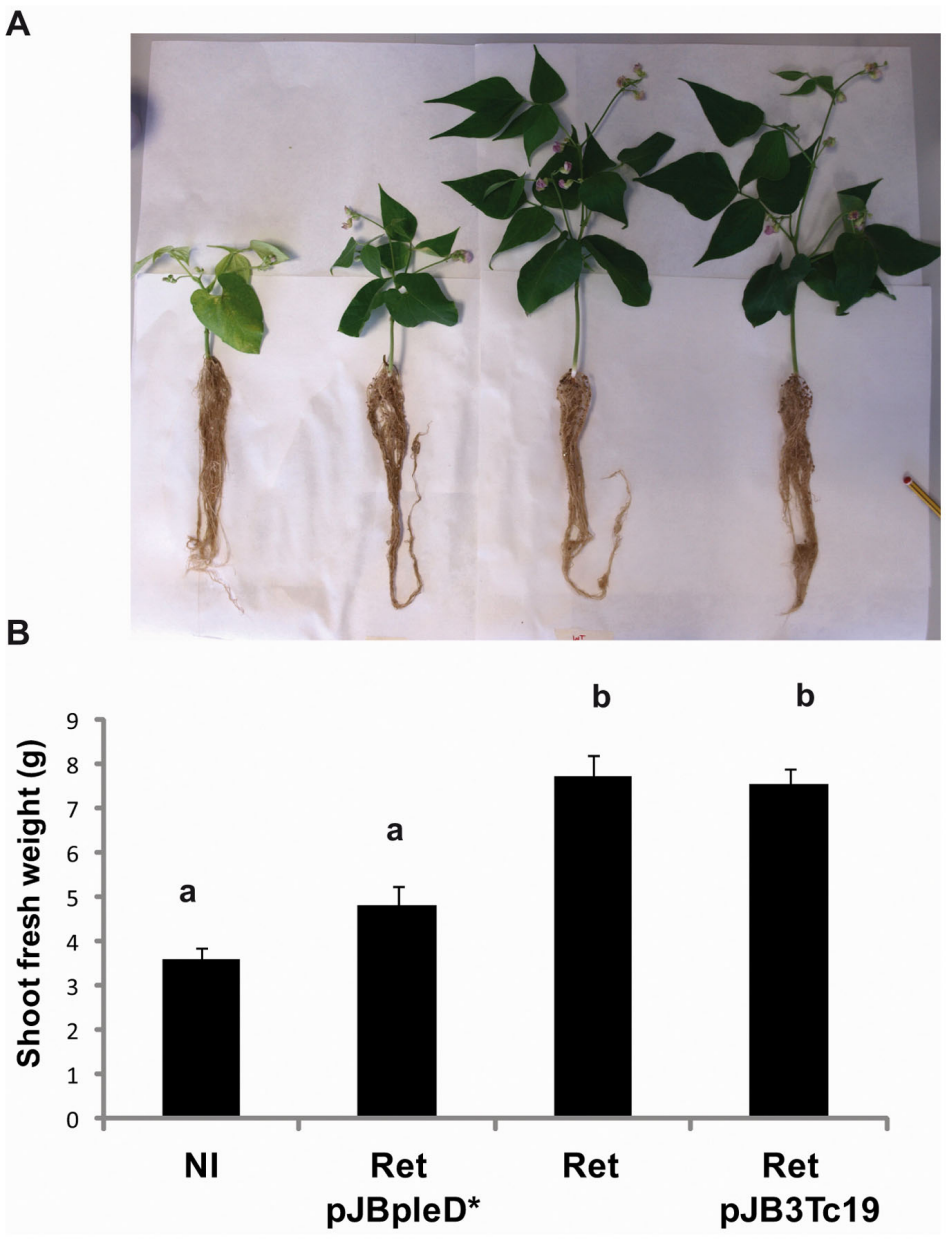
Effects of high c-di-GMP levels on the *Rhizobium etli* CFN42 (Ret) symbiotic interaction with its host. (**A**) Appearance of representative bean plants 29 days after inoculation with the indicated strains. (**B**) Mean shoot fresh weight per plant. NI, non inoculated. Bars indicate standard errors.

We tested the impact of high bacterial c-di-GMP levels in two rhizobia-legume associations forming different nodule types: *R. leguminosarum*-*Vicia sativa* and *R. etli-Phaseolus vulgaris*, that give rise to indeterminate and determinate nodules, respectively. Bacterial attachmet to roots as well as several symbiotic and plant growth related parameters were determined. Significant effects of pleD* expression were observed on both interactions, starting with enhanced attachment of bacterial cells to roots (Table 2), probably due to cellulose overproduction, since the Ret Cel^−^ mutant expressing PleD* did not exhibit that behaviour (data not shown). PleD* strains also showed a trend to form fewer nodules than the controls, although the differences were statistically significant only in the case of *R. etli*; however, average nodule size and weight seemed unaffected (Table 2). In all cases, nodule visual aspect and color were indicative of nitrogen fixation. Concerning plant parameters, aerial biomass was significantly reduced in the case of *P. vulgaris* plants inoculated with the PleD* strain (Fig. 3). In both symbioses, shoot dry weights were smaller in plants inoculated with the PleD* variants and the nitrogen content of the shoots followed a similar decreasing trend (Table 2). Concerning the stability of the pleD* construction, a strong pressure to get rid of the pJBPleD* plasmid seems to be exerted during symbiosis establishment. In the case of *P. vulgaris*, no tetracycline resistant bacteria were recovered from nodules formed by the Ret (pJBpleD*) strain (0 of 50 nodules analysed), contrasting with the 98% (49 of 50 nodules) of Ret (pJB3Tc19) nodule bacteria which maintained the tetracycline resistance vector. When plasmid stability was measured in *V. sativa* nodules, we could isolate Tc^r^ bacteria from 86% of nodules formed by the Rle strain carrying the empty vector, and from 70% of the PleD* nodules. However, it is important to remark that only one to few CFU per nodule could be recovered from most those PleD* nodules, in clear contrast with the pJB3Tc19 nodules, from which hundreds to thousands Tc^r^ CFU were usually recovered. Thus, the majority of the PleD* nodules contained none or few bacteria that still maintained the pJBpleD* plasmid. Compared to plasmid loss in nonselective media under free-living conditions (Table S2), it would seem that plasmid unstability was higher in symbiotic conditions, especially for pJBPleD*.

**Table 2:** Parameters related to the interaction of PleD* rhizobial strains with their hosts.

Plant	Rhizobia	Attachment^a^	N° of nodules^b^	Nodule weight^c^	SDW^d^	Nitrogen^e^
*Phaseolus vulgaris*	Ret pJB3Tc19	0.04±0.01%†	301±19†	3.33±0.24	0.95±0.04†	35.35±3.22†
	Ret pJBpleD*	6.55±0.73%†	203±12†	4.30±0.42	0.66±0.06†	19.64±3.29†
*Vicia sativa*	Rle pJB3Tc19	2.23±0.18%†	238±33	0.73±0.06	0.41±0.09	9.93±2.24††
	Rle pJBpleD*	6.79±0.34%†	190±15	0.65±0.12	0.30±0.02	5.66±0.40††

a Percentage of bacterial cells attached per gram of roots.

b Number of nodules per plant.

c Average nodule fresh weight, in mg.

d Average shoot dry weight per plant, in g.

e Nitrogen content per plant, in mg.

† Indicates an statistically significant difference (P≤0.05) between pJB3Tc19 and pJBpleD*.

†† Indicates an statistically significant difference (P≤0.1) between pJB3Tc19 and pJBpleD*.

### Impact of High Intracellular Levels of c-di-GMP on the Interaction of Phytopathogenic Pseudomonas with Host Plants

To study the effect of pleD* expression in Pto and Pph virulence, transformants carrying pJB3Tc19 or pJBpleD* plasmids were inoculated on 4-week old tomato plants or 2-week old bean plants, respectively, and disease symptoms were evaluated after 3, 6 and 10 days. The results showed that high intracellular levels of c-di-GMP did not have any detectable impact on the virulence of either Pto or Pph, since the strains over-expressing PleD* induced the same disease symptoms as the wild type (Fig. S6, A and B). Furthermore, *in planta* growth of Pto DC3000 carrying either of these plasmids followed a similar trend over time (Fig. S6C). We also tested the stability of plasmids pJB3Tc19 and pJBpleD* in Pto-inoculated plants, and determined that after 10 days most (67%) bacteria isolated from different plants maintained the plasmid-encoded tetracycline resistance.

Despite no changes in the virulence of Pph and Pto expressing high levels of intracellular c-di-GMP seemed apparent, we compared the expression of T3SS genes in strains with pJB3Tc19 or pJBpleD*. Expression analysis was performed by quantitative reverse-transcription polymerase chain reaction (RT-qPCR) of Pto and Pph cells growing in the T3SS inducing medium MMF. In both Pto and Pph pJBpleD* cells transcripts levels of *hrpL* (the alternative sigma factor controlling the expression of T3SS genes) and *hrpA* (encoding the protein subunits composing the T3SS pili) decreased approximately 2 fold (see Fig. S6D). Thus, there was a low but consistent reduction of T3SS gene expression by high c-di-GMP, although this seemed to have no effects on the severity of disease symptoms in their respective hosts.

**Figure 4:**
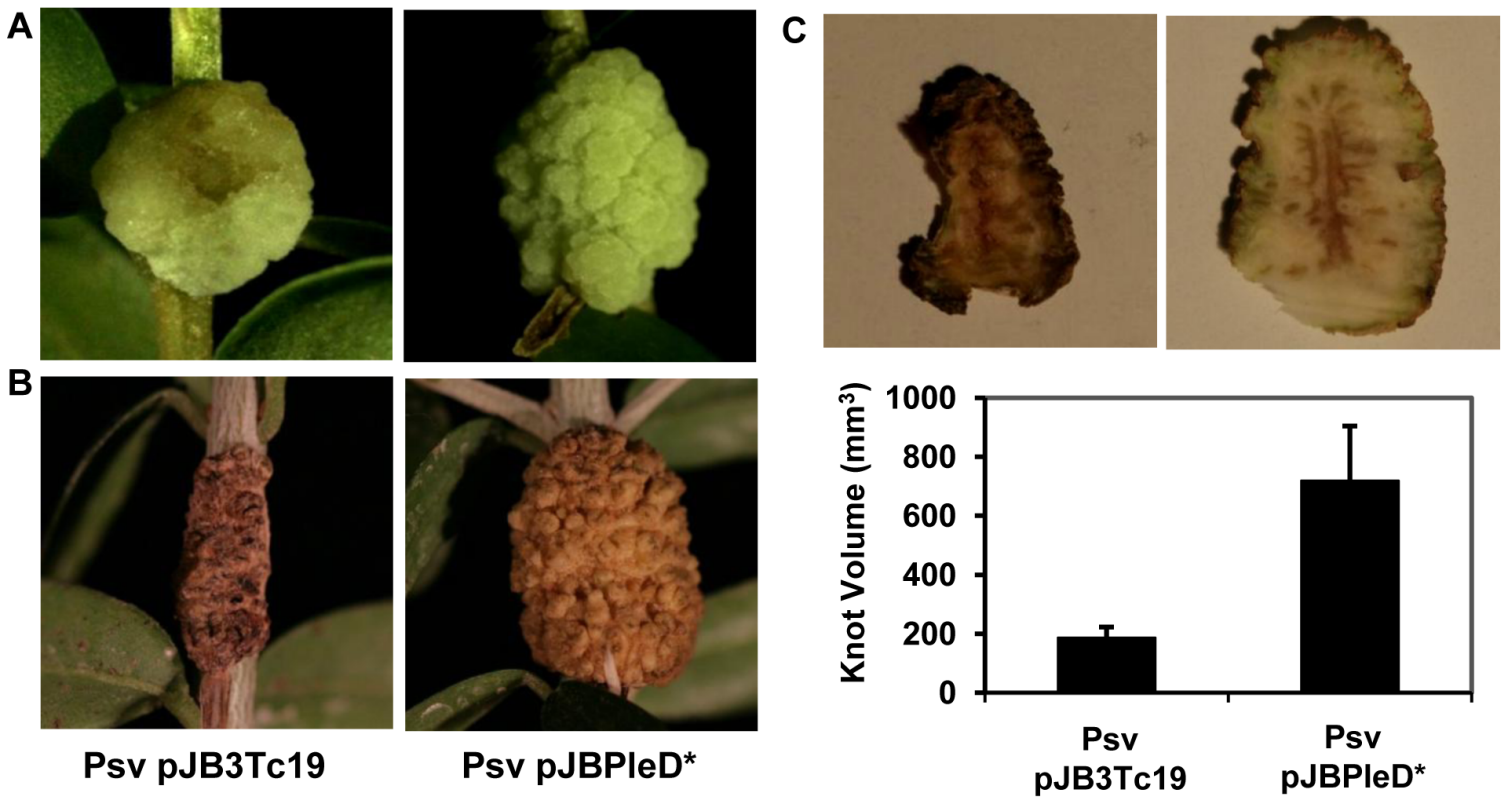
Virulence of *P. savastanoi* pv. savastanoi NCPPB 3335 expressing the pleD* gene in olive plants. Knots induced by Psv pJB3Tc19 (left), and Psv pJBPleD* (right), on young micropropagated olive plants at 30 days dpi (**A**) and in one year-old olive plants at 90 dpi (**B**). Cross sections of knots of one year-old olive plants infected with the indicate strains and their mean volumes (**C**). Knot sizes are the means of 6 different knots ± standard error.

We also examined whether expression of the pleD* gene in Psv had an effect in the development of olive knot symptoms. Psv transformants carrying plasmids pJB3Tc19 or pJBpleD* were inoculated both on *in vitro* micropropagated olive plants and on 1-year-old olive plants, and disease severity was evaluated after 30 days or 90 days, respectively. In addition, the stability of plasmid pJBpleD* was checked in Psv-infected micropropagated olive plants, and determined that from 7 dpi until the end of the experiment (30 dpi), only about 25% of the bacterial cells isolated from olive knots were resistant to tetracycline, and thus stably maintained the plasmid (Fig. S7). Despite the low stability of plasmid pJBpleD* in Psv, the results showed that expression of pleD* drastically increased knot size both on *in vitro* micropropagated (Fig. 4A) and 1-year-old olive plants (Fig. 4B). In fact, knot volume was increased significantly (P≤0.05) in the stems of one-year old olive plants inoculated with the pleD* transformant as compared with those inoculated with the control strain (Psv carrying pJB3Tc19; Fig. 4C). Conversely, transverse sections of knots induced at 90 days post-inoculation (dpi) by the pleD* transformant on 1-year-old olive plants showed a reduced necrosis of the internal tissues as compared with those developed in plants inoculated with the control strain (Fig. 4C), suggesting a delay in the maturation process of the knots induced by the pleD* transformant. Taken together, these results suggested the existence of a possible interplay between pleD*-mediated intracellular levels of c-di-GMP and the expression of both phytohormones- and type III secretion system (T3SS)-related genes in Psv. However, we could not detect significant changes in the transcript levels of *hrpL* or *hrpA* in Psv overexpressing the DGC PleD* after transfer to MMF medium, neither could we observe significant variations in transcription of gene *iaaM* (encoding triptophane monooxygenase, which converts tryptophan to indoleacetamide in the IAA biosynthetic pathway; data not shown).

## Discussion

Cyclic-di-GMP has emerged during the last decade as an ubiquitous second messenger in bacteria. However, our understanding of c-di-GMP signaling is still fragmentary probably due to the diversity of cellular functions and the great variety of mechanisms of action displayed by this dinucleotide (reviewed in [5]). To this complexity adds the fact that many bacterial genomes encode multiple proteins involved in the c-di-GMP turnover, with a special abundance among bacteria with a variety of lifestyles and coevolutionary relationships with eukaryote hosts [41]. This contrasts with the comparatively few c-di-GMP protein receptors/effectors known so far which, unlike c-di-GMP metabolizing proteins, are structurally and functionally more diverse and yet hardly identifiable through bioinformatics (with the exception of PilZ domains; [42], [43]). Thus, additional research approaches, besides genomics and bioinformatics, need to be implemented to uncover novel c-di-GMP regulation pathways, particularly in bacteria with complex lifestyles like those associating with plants. One of such approaches involves the artificial alteration of intracellular c-di-GMP levels through the expression of DGCs or PDEs in order to assess its effect on particular phenotypes. In our study, we have used the well characterized DGC PleD* to increase the levels of this second messenger in different symbiotic and phytopathogenic bacteria of great agronomic relevance. Evaluation of the free-living and plant-related behaviors clearly shows a significant impact of high c-di-GMP on both types of plant-interacting bacteria.

As it has been reported for other bacteria [42], [44], raising c-di-GMP levels causes a prominent reduction of bacterial motility, significantly changes colony morphology and increases biofilm formation in all our model strains, likely through the upregulation of extracellular polysaccharide production. Nevertheless, we spotted significant and interesting differences, as well as some novelties among the strains analyzed. Most strains seem to be able to produce c-di-GMP dependent cellulose, except Pph which lacks cellulose biosynthesis genes [45]. Cellulose is needed for c-di-GMP dependent biofilm formation by *R. etli* CFN42, since the Cel^−^ mutant did not produce it. Interestingly, we could reveal that besides cellulose, *R. etli* CFN42 produces other CR- and CF-binding c-di-GMP activated compound(s) of yet unknown nature that deserves to be characterized in a future work. Since three putative cellulose synthase genes have been annotated in its genome (http://genome.microbedb.jp/rhizobase), this strain may carry additional unknown cellulose synthesis systems; however, only the one mutated in this work resembles typical bacterial cellulose synthases [46], [47].

In contrast to the formation of strong and visually obvious archetypal surface biofilms by rhizobia, we observed the formation of c-di-GMP dependent A-L pellicles by *P. syringae* strains, suggesting a strong cell to cell attachment but loose adhesion of bacteria to the container surface (borosilicate tubes or polystyrene microtitre wells). Similar A-L interface biofilms were produced by some environmental *Pseudomonas* isolates and cellulose was proposed as the principal component of the biofilm matrix [48], [49]. However, the *P. syringae* pellicles may or may not contain cellulose, since both the wild-type Pph (which is cellulose negative) and the Pto Cel^−^ mutant were still able to form such pellicles. We found that alginate production was induced by high c-di-GMP levels in both Pto and Pph, consistent with reports for other bacteria like *P. aeruginosa* [50]. Interestingly, c-di-GMP-dependent alginate production by Pph was much higher than in the cellulose-producing strain Pto, suggesting that the lack of one EPS may be compensated by increasing the production of others. It remains to be investigated if *P. syringae* can produce, besides alginate or cellulose, additional polysaccharides under high levels of c-di-GMP (i.e., levan or psl, as described in *P. aeruginosa*; [51]) and which of those polimers is essential for pellicle formation.

One of the critical early steps in rhizobia-legume interactions is the bacterial attachment to the host plant root [2], [52]. The pleD* over-expression strongly increased the ability of Ret and Rle to attach to bean and vetch roots, respectively, most likely due to the c-di-GMP-dependent cellulose overproduction, although the participation of other type of compounds cannot be excluded. A two-steps model has been proposed for attachment of rhizobia to plant cells: the first phase involves attachment of bacteria to root hairs through the Ca^++^-binding protein rhicadhesin and/or bacterial surface polysaccharides, and the second step involves cap formation through bacterial aggregation or agglutination and requires the active synthesis of bacterial cellulose fibrils [2], [53], [54]. The fact that enhanced cellulose production of the PleD* strains correlates with incresased attachment to roots, indicates that c-di-GMP participates in the second step by activating cellulose synthesis. How cellulose gene expression is regulated in rhizobia remains unknown, albeit some authors have suggested induction by plant compounds [55], [56]. Also, cellulose-mediated aggregation has been suggested to indirectly contribute to infection by enhancing bacterial growth on the host plant root; however, cellulose-deficient mutants resulted to be as infective as Cel^+^ strains [53]–[55].

Our results also reveal important effects of c-di-GMP at other stages of the symbiotic interaction. High c-di-GMP levels strongly impaired the symbiotic efficiency of both *R. etli* and *R. leguminosarum* bv. viciae, as evidenced by the reduced shoot weights and N contents of the plants inoculated with PleD* strains. Nodule number was also significantly reduced in the case of *R. etli*-*P. vulgaris* but not in the *R. leguminosarum*-vetch interaction. We need to point out that, although nodules formed by the PleD* strains had normal size and appearance, contained few o none bacteria or bacteroids carrying the PleD* plasmid, therefore they mainly contained wild-type like bacteria and bacteroids by the end of the experiment. The high intracellular c-di-GMP levels appear to have imposed a strong pressure against progression of the symbiosis, which might not have proceeded until a significant loss of the pleD* plasmid occurred within the inoculant population. Plasmid loss could have happened during bacterial multiplication in the rhizosphere and rhizoplane or afterwards during nodule induction and invasion. Hence, high c-di-GMP levels likely determine a delay of symbiosis establishment, although it is difficult to discern if one or more stages (e.g., nodule induction, nodule infection, bacteroid differentiation or function) are directly affected. Therefore, it will be of great interest to assess the impact of the c-di-GMP economy at various (early and late) stages of symbiosis establishment using more stable, i.e. chromosomal, PleD* constructions in which high c-di-GMP levels will be maintained throughout symbiosis development.

High c-di-GMP levels in phytopathogenic *Pseudomonas* had varying effects on the development of disease symptoms. On one hand, Pto and Pph overexpressing PleD* did not show any changes in virulence, despite important phenotypes like motility or EPS production were clearly altered. Among the overproduced EPSs was alginate, which has been associated with phenotypes important for virulence [57], [58]. Thus, it is possible that c-di-GMP positively impacts on certain traits (i.e., alginate production) compensating for the negative effects on other traits (T3SS gene transcription), leading to an apparent lack of visual differences in disease symptoms. Conversely, high levels of c-di-GMP in Psv resulted in an increased knot size on olive plants. However, we did not observe significant changes in *iaaM* gene transcription, suggesting that besides IAA production, additional bacterial traits may also affect knot size. In fact, knot induction by Psv-infected plants has been shown to be dependent on several other factors, including a putative DGC [59]. Although increased knot size could be seen as an increased virulence of the strain, a detailed inspection of knots showed a reduced necrosis of the internal tissues (Fig. 4C), suggesting a delay in the maturation of the knots induced by the Psv pleD* transformant. Regulation of virulence by c-di-GMP in bacterial phytopathogens belonging to the *P. syringae* complex, which includes *P. savastanoi,* has not been reported to date. However, c-di-GMP has been shown to negatively regulate *hrpL* and *hrpA* transcription in the bacterial phytopathogen *D. dadantii*, through a mechanism probably mediated by RpoN [60]. Also, an artificial increase in c-di-GMP levels in *P. aeruginosa*, upon overproduction of the DGC WspR, has been shown to control the levels of T3SS and T6SS proteins in an inverse manner [61]. In contrast to the *P. syringae* strains, we could not observe significant effects of high c-di-GMP on T3SS gene transcription of *P. savastanoi*, although disease features were clearly affected, suggesting that c-di-GMP could regulate the Psv T3SS in a different manner. In fact, different c-di-GMP regulatory mechanisms have been demonstrated in bacteria [61], including transcriptional regulation [62], translational regulation [63], regulation of protein activity [64] and cross-envelope signalling [65].

In summary, an increment of the intracellular levels of c-di-GMP seems to promote a plant-associated life-style *versus* a saprophytic one by reducing the motility, increasing EPS production and promoting biofilm formation. However, several reports have evidenced a negative regulation of virulence by c-di-GMP in plant pathogens like *Xanthomonas campestris* [16], [66], *Dickeya dadantii* [60], *Pectobacterium atrosepticum* [67], [68], *Agrobacterium tumefaciens* [69], [70] or *Erwinia amylovora* [71]. The case of *Xylella fastidiosa,* where high c-di-GMP increases virulence, seems to be unique [72]. In several plant beneficial bacteria, c-di-GMP positively regulates important features for rhizosphere colonization, like in *Pseudomonas putida* [73], *P. fluorescens* [74], [75] or *Rhizobium* [76].

Our results show that, for a given plant-bacterial system, high levels of c-di-GMP provoke contrasting effects depending on the stage of the interaction, e.g. promoting rhizobial attachment to the legume root but impairing symbiosis establishment, or increasing the volume of knots induced in olives by Psv whereas reducing necrosis of plant tissues. This suggests that there is a need of fine tuning the c-di-GMP intracellular levels to ensure the stage transition during the establishment of the association with the host.

## Supporting Information

Figure S1 **Intracellular c-di-GMP contents.** c-di-GMP in cell extracts of *Rhizobium etli* CFN42 (Ret), *Rhizobium leguminosarum* bv. viciae UPM791 (Rle), *Pseudomonas savastanoi* pv. savastanoi NCPPB 3335 (Psv), *Pseudomonas syringae* pv. tomato DC3000 (Pto) and *Pseudomonas syringae* pv. phaseolicola 1448 (Pph) expressing pleD* (pJBpleD*) and their respective control strains (pJB3Tc19, empty vector). Values are the means of 3 biological replicates ± standard error. See Material and Methods for details.
(TIF)

Figure S2 **Bacterial growth curves.** Growth curves of Pto and Ret Cellulose synthase mutants carrying plasmid pJB3Tc19 or pJBPleD*.
(TIF)

Figure S3 **Calcofluor-derived fluorescence.** Quantification of calcofluor-derived fluorescence of cultures of *Rhizobium etli* CFN42 (Ret) and its Cel^−^ mutant derivative, *Rhizobium leguminosarum* bv. viciae UPM791 (Rle), *Pseudomonas savastanoi* pv. savastanoi NCPPB 3335 (Psv), *Pseudomonas syringae* pv. tomato DC3000 (Pto) and its Cel^−^ mutant derivative, and *Pseudomonas syringae* pv. phaseolicola 1448 (Pph) expressing pleD* (pJBpleD*) and their respective control strains (pJB3Tc19, empty vector). Mean values from 3 independent cultures ± standard error.
(TIF)

Figure S4 **Quantification of alginate production.** Alginate production in *Pseudomonas syringae* pv. tomato DC3000 (Pto) and *Pseudomonas syringae* pv. phaseolicola 1448 (Pph) expressing pleD* (pJBpleD*) and their respective control strains (pJB3Tc19, empty vector). Values are the means of 5 independent replicates ± standard deviation.
(TIF)

Figure S5 **Motility and high c-di-GMP. Effects of high c-di-GMP levels on bacterial motility.** *Pseudomonas syringae* pv. phaseolicola 1448 (Pph) and *Rhizobium leguminosarum* bv. viciae UPM791 (Rle) are shown as representative strains. **(A)** Swimming tests in Bromfield medium (0.3% agar) supplemented with tetracycline after 3 days at 28°C for Rle strains and in 0,3% LB agar plates supplemented with tetracycline after 2 days at 25°C for Pph strains. **(B)** Surface motility on semisolid MM plates (0.6% agar) 3 days after inoculation at 28°C for Rle and in PG-agar plates (0,5%) 24 hours after inoculation at 25°C for Pph. Similar results were obtained for the rest of strains, except Psv which did not show surface motility in any condition tested.
(TIF)

Figure S6 **Virulence of** ***Pseudomonas syringae*** **.** Disease symptoms in leaves of common bean at 6 dpi (**A**) or tomato at 9 dpi (**B**) induced by *Pseudomonas syringae* pv. phaseolicola 1448 (Pph) or *Pseudomonas syringae* pv. tomato DC3000 (Pto), respectively, expressing pleD* (pJBpleD*) and their respective control strains (pJB3Tc19, empty vector). (C) Bacterial growth on tomato leaves. Time course of *in planta* growth of Pto DC3000 pJB3Tc19 (black), and Pto DC3000 pJBpleD* (white). Development of CFU on the primary leaves of tomato plants at 0, 3, 6 and 10 days after spray inoculation with approximately 10^6^ CFU/ml. Data represent the average of six experiments. (**D**) Relative transcript leves of T3SS genes *hrpL* and *hrpA* in Pto and Pph expressing pleD* (filled bars) and they respective control strains with pJB3Tc19 (empty bars); results shown are the means and standard deviations of three experiments with three replicates.
(TIF)

Figure S7 **Plasmid stability in** ***P. savastanoi*** **.** Maintenance of plasmid pJBpleD* on young micropropagated olive plants. Counts of CFU/mL in LB medium with tetracycline (triangles) and without tetracycline (circles) at 0, 7, 15, 20 and 30 dpi. Each point is the mean of three replicates. Error bars represent the standard error.
(TIF)

Table S1 **Primers used in this work.**
(DOCX)

Table S2 **Plasmid stability in nonselective medium.**
(DOCX)
