# Supplementary figures and images for: Responses to Elevated c-di-GMP Levels in Mutualistic and Pathogenic Plant-Interacting Bacteria
Source: PLoS One. 2014 Mar 13;9(3):e91645. doi: 10.1371/journal.pone.0091645 (PMC3953490; doi:10.1371/journal.pone.0091645)

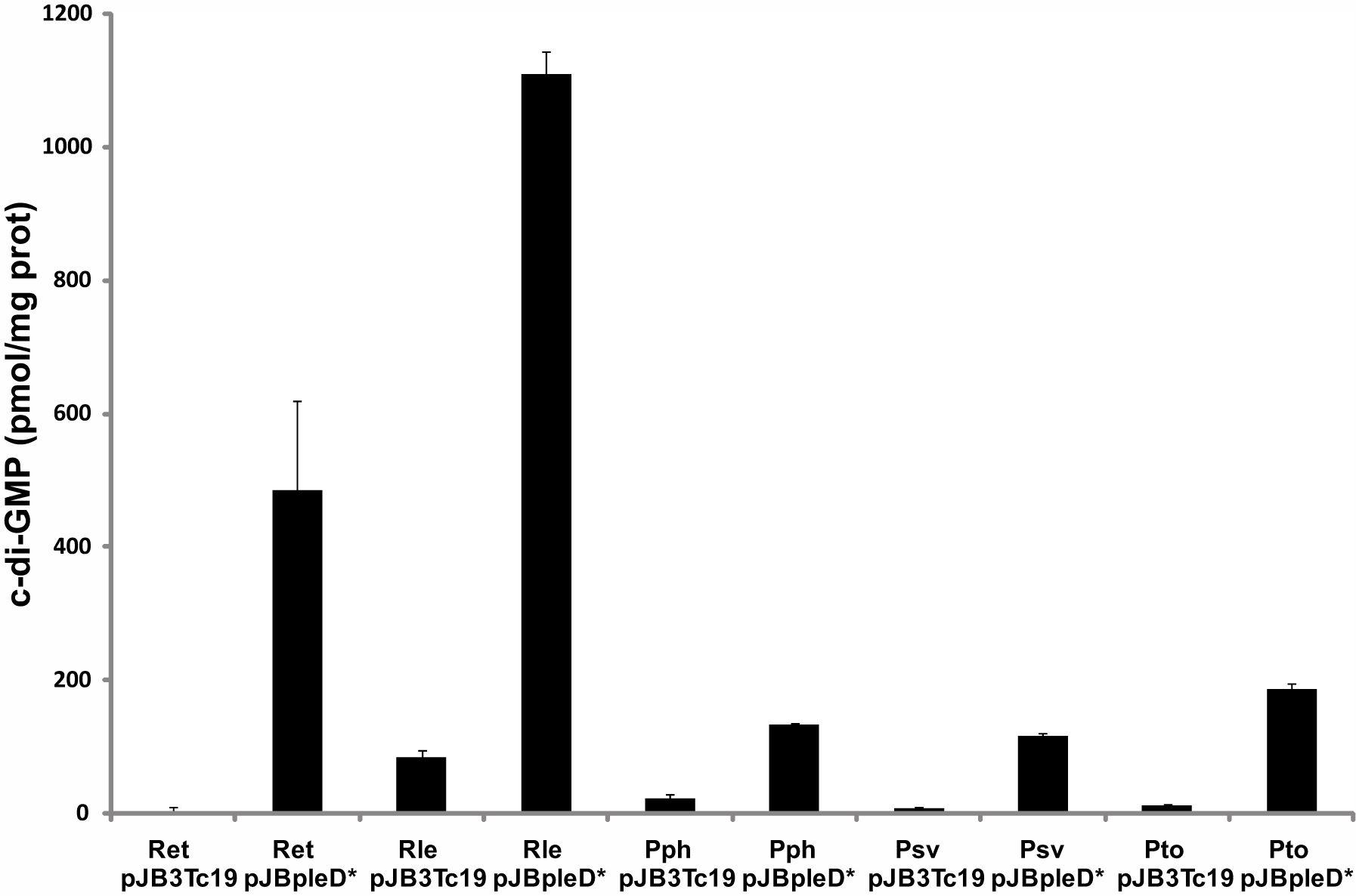

Supplement: Figure S1 — Intracellular c-di-GMP contents. c-di-GMP in cell extracts of Rhizobium etli CFN42 (Ret), Rhizobium leguminosarum bv. viciae UPM791 (Rle), Pseudomonas savastanoi pv. savastanoi NCPPB 3335 (Psv), Pseudomonas syringae pv. tomato DC3000 (Pto) and Pseudomonas syringae pv. phaseolicola 1448 (Pph) expressing pleD* (pJBpleD*) and their respective control strains (pJB3Tc19, empty vector). Values are the means of 3 biological replicates ± standard error. See Material and Methods for details. (TIF) [file pone.0091645.s001.tif]

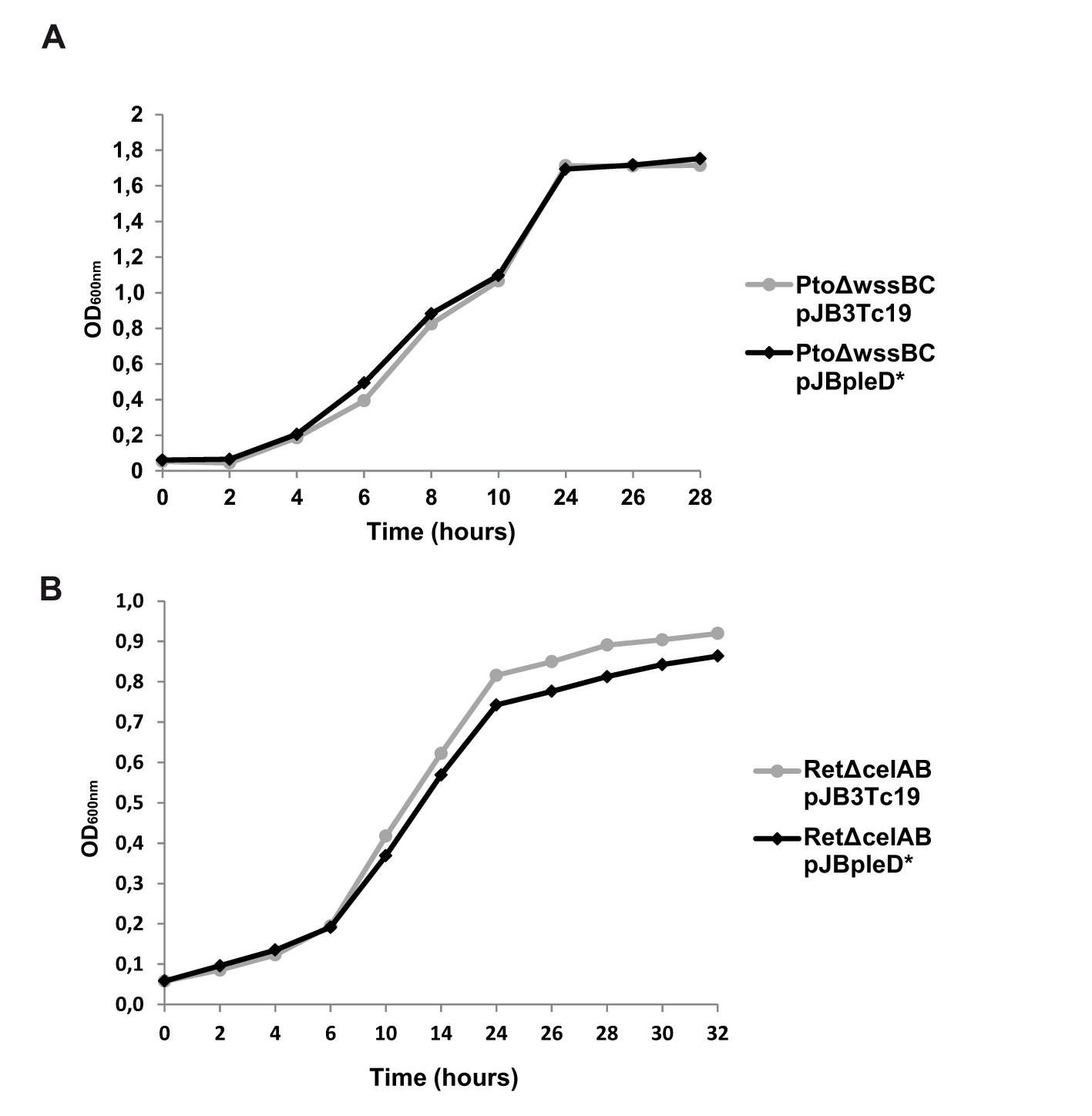

Supplement: Figure S2 — Bacterial growth curves. Growth curves of Pto and Ret Cellulose synthase mutants carrying plasmid pJB3Tc19 or pJBPleD*. (TIF) [file pone.0091645.s002.tif]

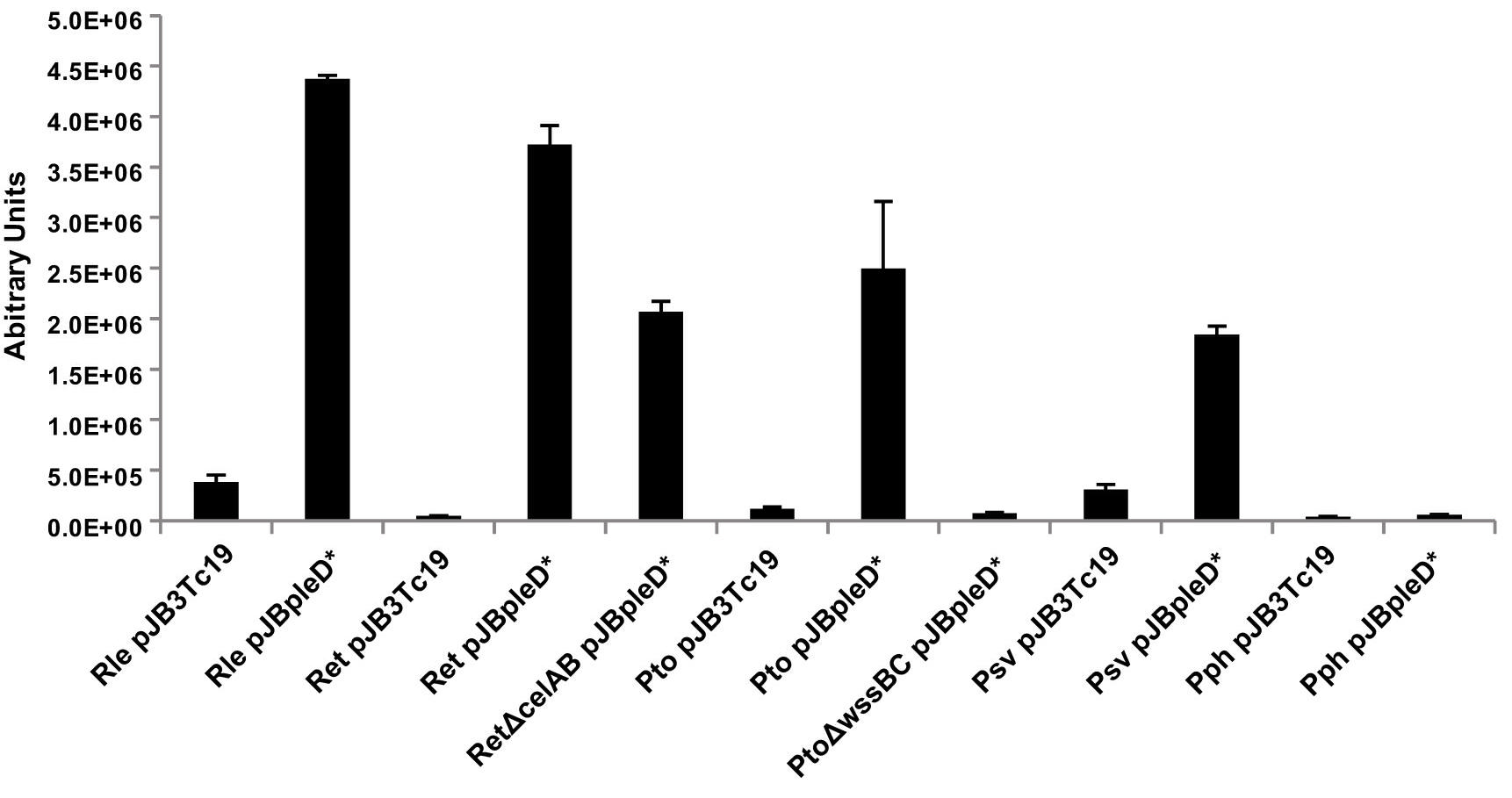

Supplement: Figure S3 — Calcofluor-derived fluorescence. Quantification of calcofluor-derived fluorescence of cultures of Rhizobium etli CFN42 (Ret) and its Cel− mutant derivative, Rhizobium leguminosarum bv. viciae UPM791 (Rle), Pseudomonas savastanoi pv. savastanoi NCPPB 3335 (Psv), Pseudomonas syringae pv. tomato DC3000 (Pto) and its Cel− mutant derivative, and Pseudomonas syringae pv. phaseolicola 1448 (Pph) expressing pleD* (pJBpleD*) and their respective control strains (pJB3Tc19, empty vector). Mean values from 3 independent cultures ± standard error. (TIF) [file pone.0091645.s003.tif]

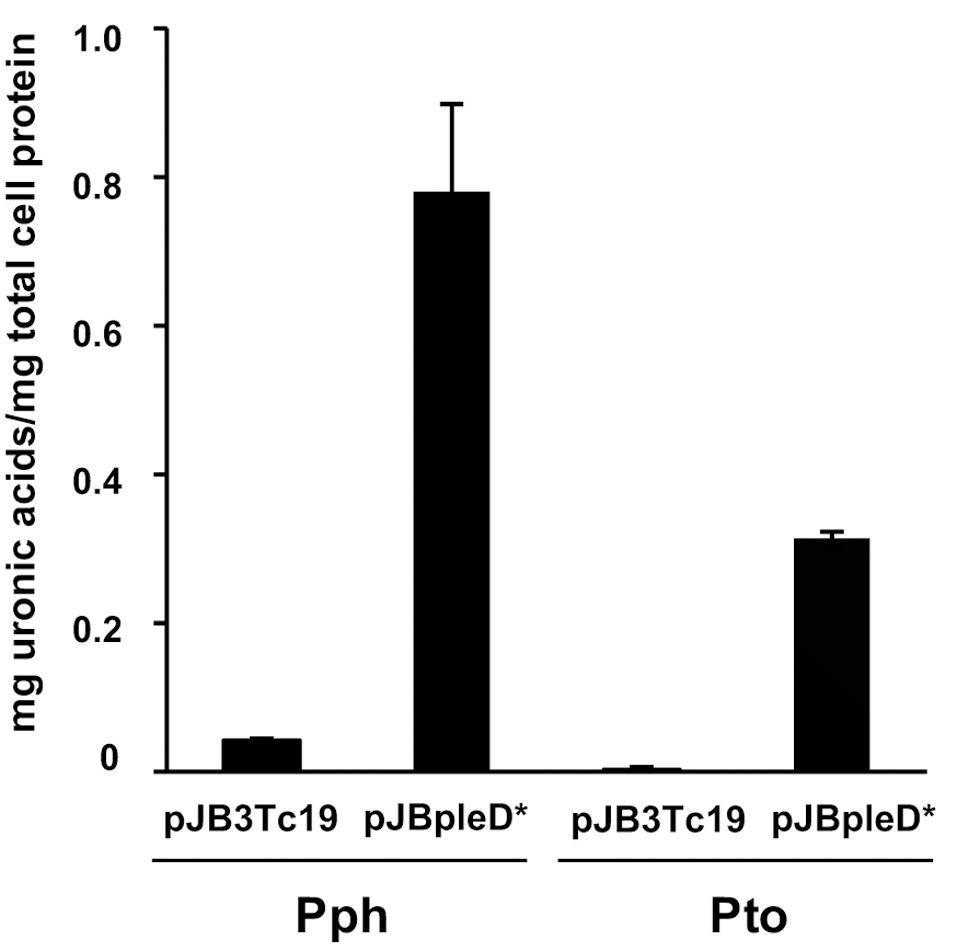

Supplement: Figure S4 — Quantification of alginate production. Alginate production in Pseudomonas syringae pv. tomato DC3000 (Pto) and Pseudomonas syringae pv. phaseolicola 1448 (Pph) expressing pleD* (pJBpleD*) and their respective control strains (pJB3Tc19, empty vector). Values are the means of 5 independent replicates ± standard deviation. (TIF) [file pone.0091645.s004.tif]

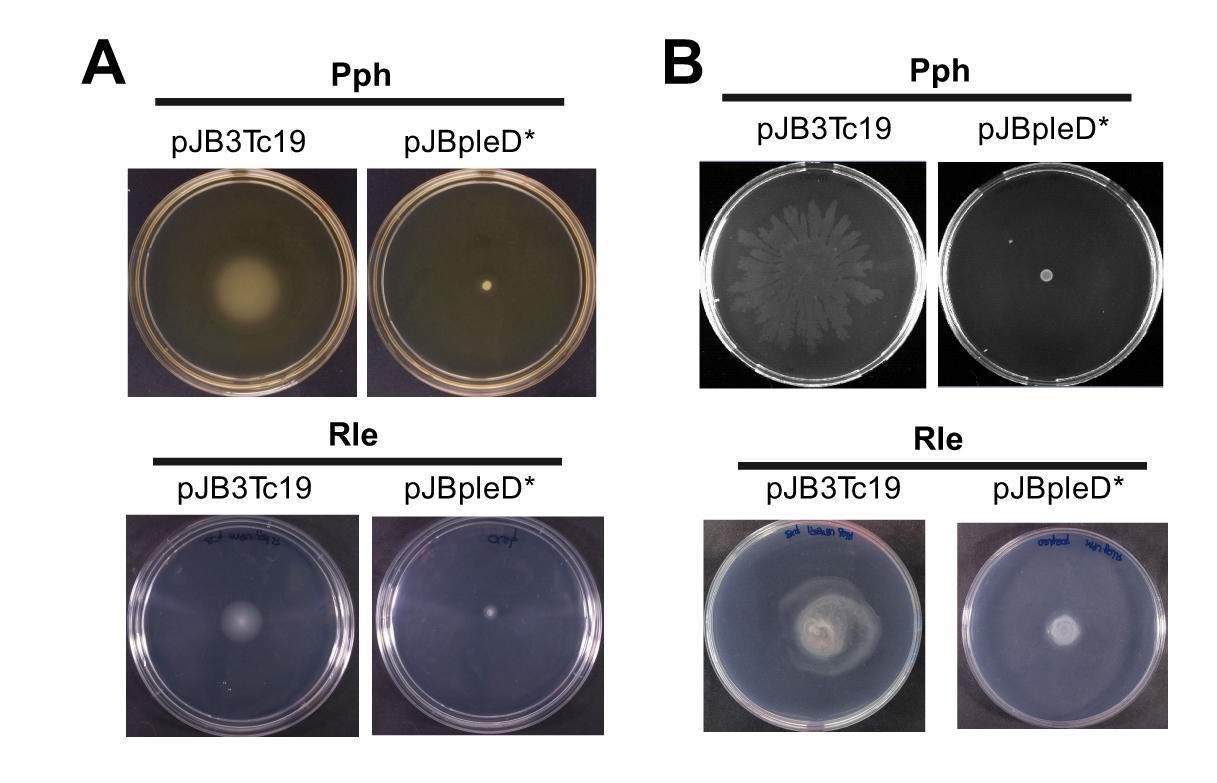

Supplement: Figure S5 — Motility and high c-di-GMP. Effects of high c-di-GMP levels on bacterial motility. Pseudomonas syringae pv. phaseolicola 1448 (Pph) and Rhizobium leguminosarum bv. viciae UPM791 (Rle) are shown as representative strains. (A) Swimming tests in Bromfield medium (0.3% agar) supplemented with tetracycline after 3 days at 28°C for Rle strains and in 0,3% LB agar plates supplemented with tetracycline after 2 days at 25°C for Pph strains. (B) Surface motility on semisolid MM plates (0.6% agar) 3 days after inoculation at 28°C for Rle and in PG-agar plates (0,5%) 24 hours after inoculation at 25°C for Pph. Similar results were obtained for the rest of strains, except Psv which did not show surface motility in any condition tested. (TIF) [file pone.0091645.s005.tif]

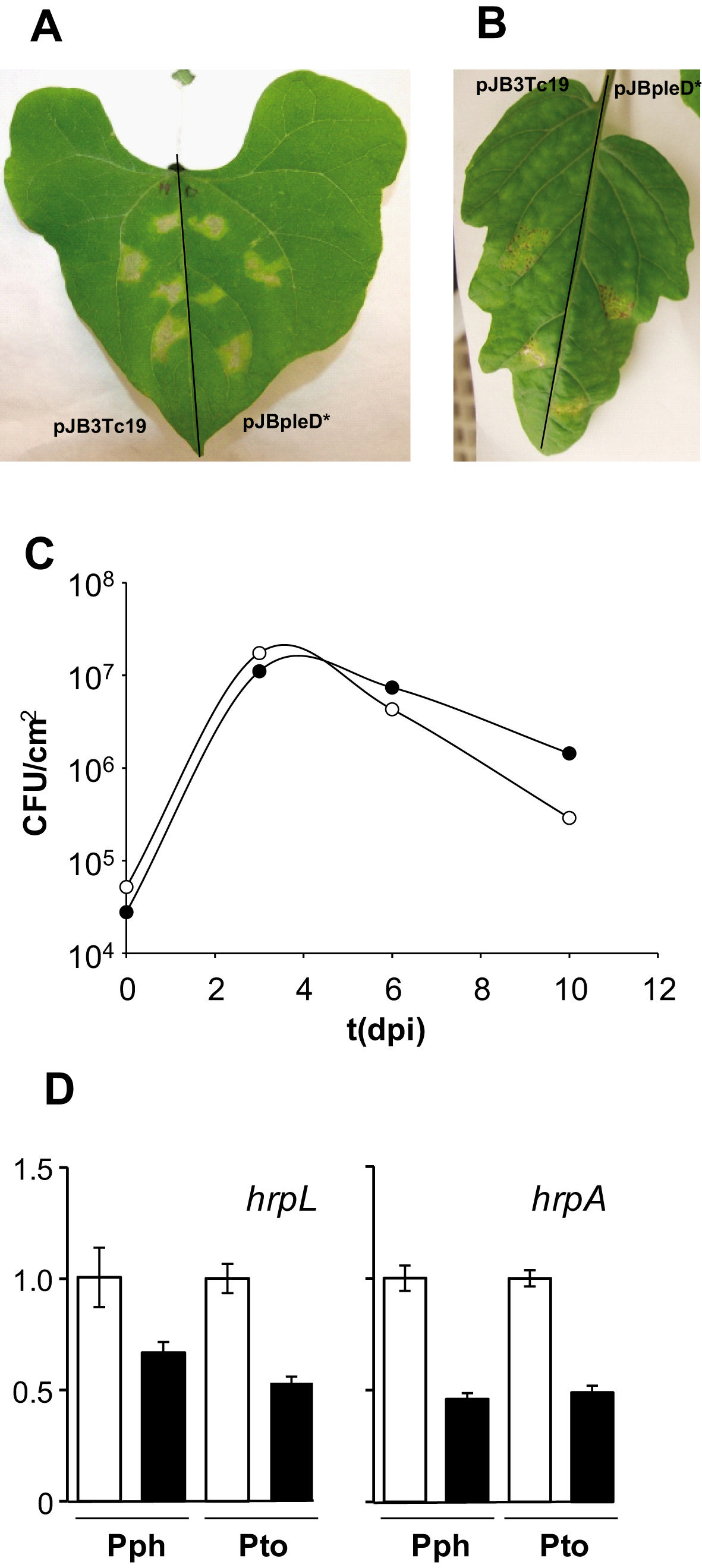

Supplement: Figure S6 — Virulence of Pseudomonas syringae . Disease symptoms in leaves of common bean at 6 dpi (A) or tomato at 9 dpi (B) induced by Pseudomonas syringae pv. phaseolicola 1448 (Pph) or Pseudomonas syringae pv. tomato DC3000 (Pto), respectively, expressing pleD* (pJBpleD*) and their respective control strains (pJB3Tc19, empty vector). (C) Bacterial growth on tomato leaves. Time course of in planta growth of Pto DC3000 pJB3Tc19 (black), and Pto DC3000 pJBpleD* (white). Development of CFU on the primary leaves of tomato plants at 0, 3, 6 and 10 days after spray inoculation with approximately 106 CFU/ml. Data represent the average of six experiments. (D) Relative transcript leves of T3SS genes hrpL and hrpA in Pto and Pph expressing pleD* (filled bars) and they respective control strains with pJB3Tc19 (empty bars); results shown are the means and standard deviations of three experiments with three replicates. (TIF) [file pone.0091645.s006.tif]

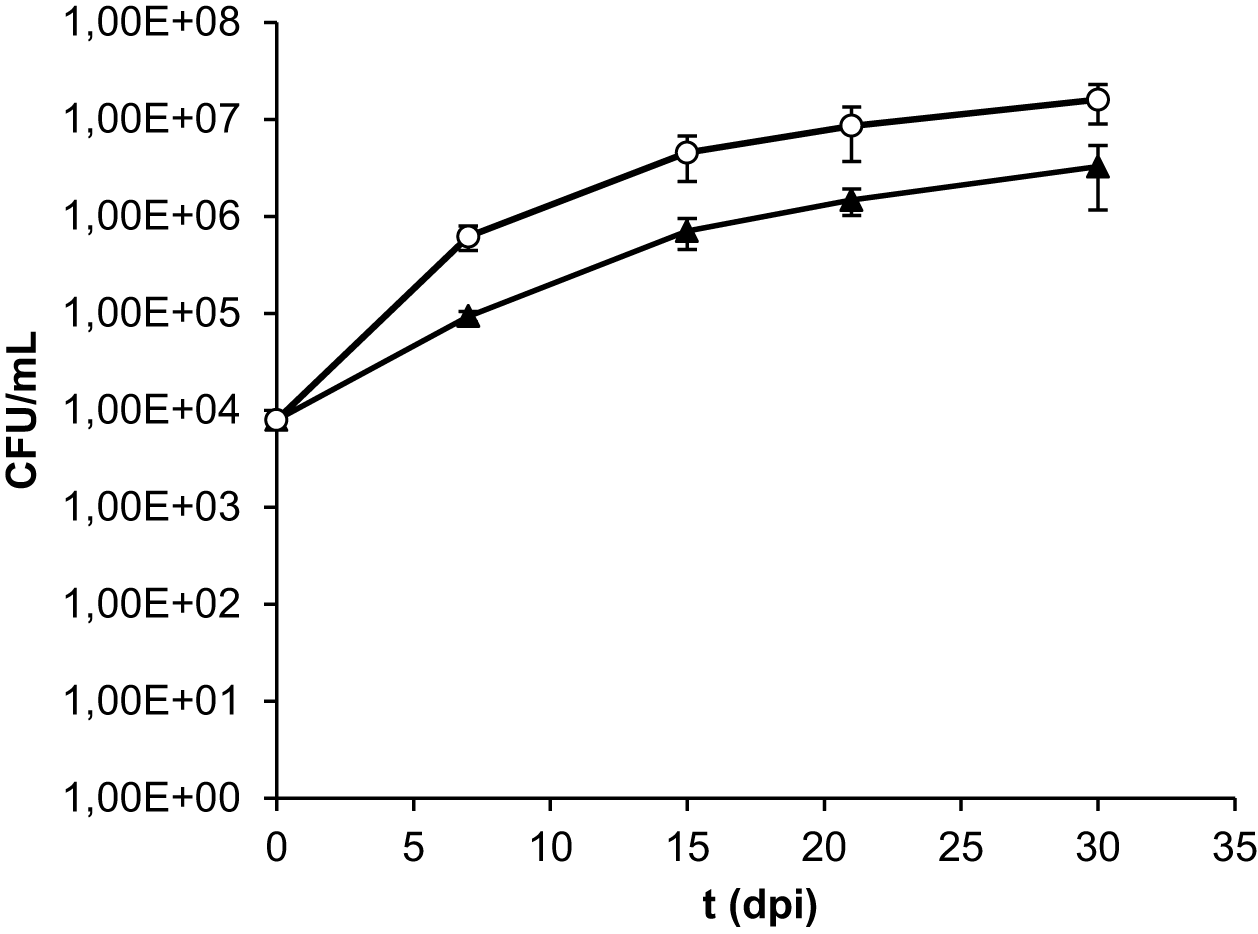

Supplement: Figure S7 — Plasmid stability in P. savastanoi . Maintenance of plasmid pJBpleD* on young micropropagated olive plants. Counts of CFU/mL in LB medium with tetracycline (triangles) and without tetracycline (circles) at 0, 7, 15, 20 and 30 dpi. Each point is the mean of three replicates. Error bars represent the standard error. (TIF) [file pone.0091645.s007.tif]
