# Supplementary material for: Responses to Elevated c-di-GMP Levels in Mutualistic and Pathogenic Plant-Interacting Bacteria
Source: PLoS One. 2014 Mar 13;9(3):e91645. doi: 10.1371/journal.pone.0091645 (PMC3953490; doi:10.1371/journal.pone.0091645)
Supplement: Table S1 — Primers used in this work. (DOCX) [file pone.0091645.s008.docx]

Table S1. Primers used in this work

| **Primer** | **Product (bp)** | **Sequence (5’ to 3’)** | **Used in** |
| --- | --- | --- | --- |
|  |  |  |  |
| Pph-GyrA-F | 135 | CAGCGTCCGCTTGCAGGTCTTCACC | qRT-PCR |
| Pph-GyrA-R |  | CGTGTTCAGGAGCCGTCGGAAGTCG |  |
|  |  |  |  |
| Pph-HrpA-F | 192 | GCGCGGCGTCGTTAACACAGTGG | qRT-PCR |
| Pph-HrpA-R |  | AGGCCGTTCTCTTCGTTCGCAGTGC |  |
|  |  |  |  |
| Pph-HrpL-F | 164 | CAAGCCGCAGACCTGGTTGTGTGG | qRT-PCR |
| Pph-HrpL-R |  | CCAATTGCCTGTGCCCGTCTACCTGA |  |
|  |  |  |  |
| Pto-GyrA-F | 205 | GGCAAAGTGACCCGCTTCAAGGAATC | qRT-PCR |
| Pto-GyrA-R |  | TGAGGATCTGGCTGCCTTCTTCCGG |  |
|  |  |  |  |
| Pto-HrpA-F | 158 | TGCACAAGCCAAGGCCAGTAAAGAGTCC | qRT-PCR |
| Pto-HrpA-R |  | TCGCTGTGGCACTGATCTTCTTGTTGGT |  |
|  |  |  |  |
| Pto-HrpL-F | 138 | CAAACCGCAGACCTGGCTGTGTGG | qRT-PCR |
| Pto-HrpL-R |  | TGACTGACATCACCGTGCCCTTCCA |  |
|  |  |  |  |
| 1026-F | 1192 | CGACACGCAGTTGTTGGC | Deletion of *wssBC* |
| 1027-R |  | GCCAGTTCGCCTGCTCGCAGAACTCGC |  |
| 1028-F | 1311 | GCGAGCAGGCGAACTGGCTGCCGAGTG |  |
| 1029-R |  | CGGTCATCCAGCCAACAG |  |
|  |  |  |  |
| CelAB-1 | 836 | AATCGGCAAGAGCAATCG | Deletion of *celAB* |
| CelAB-2 |  | ATGGCGTCCGACGGCAATGAGTTCGC |  |
| CelAB-3 | 969 | ATTGCCGTCGGACGCCATCTTCATCGG |  |
| CelAB-4 |  | GTGCGGCTTGATGTTGG |  |
|  |  |  |  |
