# Supplementary material for: Responses to Elevated c-di-GMP Levels in Mutualistic and Pathogenic Plant-Interacting Bacteria
Source: PLoS One. 2014 Mar 13;9(3):e91645. doi: 10.1371/journal.pone.0091645 (PMC3953490; doi:10.1371/journal.pone.0091645)
Supplement: Table S2 — Plasmid stability in nonselective medium. (DOCX) [file pone.0091645.s009.docx]

Table S2. Plasmid stability in nonselective medium.

|  | **% Plasmid maintenance^1^** | |
| --- | --- | --- |
| **Bacterial Strain** | pJB3Tc19 | pJBPleD* |
| Pto DC3000 | 41% | 45% |
| Pph 1448A | 100% | 90% |
| Psv NCPPB 3335 | 83% | 87% |
| Rle UPM791 | 73.5% | 67% |
| Ret CFN42 | 8.3% | 0.6% |

^1^ Percentage of CFU that maintained the plasmid-encoded Tc resistance after approximately 100 generations.
